# Impact of Methamphetamine Abuse: A Rare Case of Rapid Cerebral Aneurysm Growth with Review of Literature

**DOI:** 10.1155/2018/1879329

**Published:** 2018-10-04

**Authors:** James Fowler, Brian Fiani, Syed A. Quadri, Vladimir Cortez, Mudassir Frooqui, Atif Zafar, Fahad Shabbir Ahmed, Asad Ikram, Anirudh Ramachandran, Javed Siddiqi

**Affiliations:** ^1^Department of Neurosurgery, Desert Regional Medical Center, Palm Springs, CA, USA; ^2^Department of Neurology, University of New Mexico, Albuquerque, NM, USA; ^3^Department of Pathology, Yale School of Medicine, New Haven, CT, USA; ^4^College of Osteopathic Medicine of the Pacific, Western University of Health Sciences, Pomona, CA, USA

## Abstract

Methamphetamine or “meth” is a sympathomimetic amine of the amphetamine-type substances (ATS) class with an extremely high potential for abuse. Illicitly abused neurostimulants like cocaine and meth predispose patients to the aneurysmal formation with reported rupture at a younger age and in much smaller sized aneurysms. However, very rapid growth of aneurysm within less than 2 weeks with methamphetamine abuse is very rarely observed or reported. In this report, we present a patient with repeated and recurrent meth abuse who demonstrated rapid growth of a pericallosal aneurysm over the period of less than two weeks. The pathophysiology of stroke related to meth and ATS abuse is multifactorial with hypertension, tachycardia, and vascular disease postulated as major mechanisms. The rapid growth of an aneurysm has a high risk of aneurysmal rupture and SAH, which is a neurosurgical emergency and therefore warrants careful consideration and close monitoring. This case confirms the dynamic temporal effects of methamphetamine use on intracranial vessels and this specific neurostimulants association to rapid aneurysmal formation. In light of vascular pathologies the possibility of drug-induced pseudoaneurysm should also be considered in young patients with history of meth abuse.

## 1. Introduction

Methamphetamine, commonly known as “meth”, is a sympathomimetic amine of the amphetamine-type substances (ATS) class with an extremely high potential for abuse [1]. This abuse potential comes from side effects like euphoria, hallucination, central nervous system (CNS) stimulation, and anorexia. Excess use of meth has been implicated in the formation of renal and splanchnic artery aneurysms [2] and may cause myocardial infarction or stroke [3, 4]. Neurostimulants like cocaine and meth predispose patients to the aneurysmal formation with reported rupture at an earlier age and in much smaller sized aneurysms [4–7]

In this report, we present a patient with repeated and recurrent meth abuse who demonstrated rapid growth of a pericallosal aneurysm over the period of fewer than two weeks. Such rapid growth of aneurysm in the major intracranial vessels resulting from methamphetamine abuse is very rare. The pathophysiology of stroke form ATS Class substances is multifactorial but preexisting vascular disease, and meth-induced hypertension pays a major role [8]. The rapid growth of an aneurysm has a high risk of aneurysmal rupture and SAH, which is a neurosurgical emergency [3, 9]. Some underlying risk factors have been identified which can be helpful in recognizing susceptible individuals, which can benefit in clinical decision-making [10, 11].

## 2. Case

**Figure 1:**
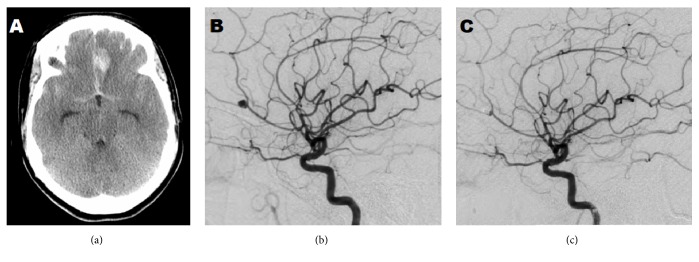
Imaging study upon patient's arrival at the hospital in 2012. (a) CT head without contrast showing diffuse subarachnoid hemorrhage that originated from an aneurysm identified by (b) Cerebral angiogram. (c) Cerebral angiogram imaging after coiling of an aneurysm.

A 41-year-old Hispanic woman initially presented to the emergency room (ER) in 2012 with a severe excruciating headache for approximately 1 hour after the use of meth. Further history revealed patient had been an oral, snorting and intravenous user of meth. Her headache was also associated with nausea, vomiting, neck pain/stiffness, and photophobia. The patient had the following vitals: blood pressure 146/94 mmHg, heart rate 64 beats/min, respiratory rate 18 breath/min, and Temperature 36.5°C. The further patient assessment revealed a Hunt and Hess: grade I (+1) and Glasgow Coma Scale (GCS) of 15 with no focal deficits. Blood workup (hematological and blood chemistry) was within normal range. A head CT demonstrated left frontal intraparenchymal hemorrhage (IPH) measuring 1.2 × 2.6 cm with bilateral frontal and Sylvian fissure subarachnoid hemorrhage (Figure 1(a)) with hemorrhagic extension into the fourth ventricle; Fisher grade: IV. CT-A demonstrated a left distal anterior cerebral artery aneurysm measuring 3.7 × 3.4 mm pointing in a superior-medial direction. An EVD was placed for obstructive hydrocephalus. After coiling the ruptured aneurysm, postcoiling images were also obtained (Figure 1(c)). After procedure the patient was stable and without neurological deficit; her ICU stay was uneventful and eventually discharged home.

**Figure 2:**
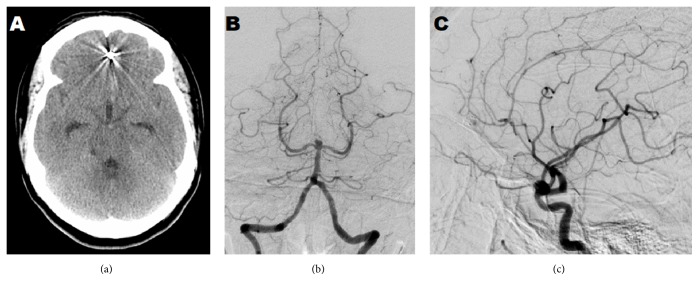
Imaging upon hospitalization in 2016. (a) CT head without contrast in 2016 showing intracerebral hemorrhage adjacent to the previously coiled aneurysm. (b and c) Cerebral angiogram of the anterior and posterior circulations showing the development of multiple new aneurysms.

The patient was lost to follow-up and presented in the hospital emergency room after four years with complaints of acute onset headache similar to prior presentation with headache and vomiting identical to the symptoms she had in 2012. Follow-up history revealed she continued to struggle with meth abuse with last use around ten days before her presentation to the hospital. Her vitals on presentation were as follows: blood pressure 129/54 mmHg, heart rate 61 beats/min, respiratory rate 16 breath/min, and Temperature 37°C. The patient was assessed as Hunt and Hess grade II + 1 and a GCS of 15 with no focal deficits. There was a left medial frontal intracerebral hemorrhage adjacent to an aneurysm that was coiled previously on head CT (Figure 2(a)). A formal angiogram demonstrated rerupture with recannulation at the base of the previously coiled aneurysm. Additionally, three neoaneurysms that were found include a periophthalmic aneurysm of the right internal carotid artery, approximately 5 mm, a fusiform dilation aneurysm at the pericallosal and callosomarginal bifurcation, and a basilar tip aneurysm, approximately 1.5-2 mm and 4 mm, respectively (Figures 2(b) and 2(c)) and were assessed as Fisher grade IV. She subsequently underwent a bifrontal craniotomy for clipping of her complex anterior cerebral branch aneurysm. Her postoperative course was uneventful, and the patient was eventually discharged home, GCS 15, and neurologically intact.

**Figure 3:**
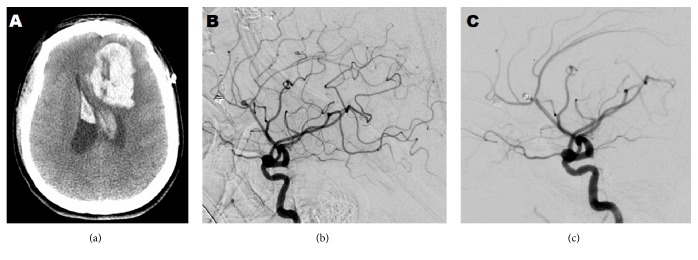
(a) 5 days after discharge in 2016: CT head shows large intracerebral hematoma. (b) A diagnostic cerebral angiogram less than 3 weeks from previous imaging demonstrating interval development of a saccular aneurysm at the callosomarginal and pericallosal bifurcation. (c) Cerebral angiogram postcoiling.

Five days after discharge in 2016, the patient again presented to the hospital as a transfer from an outside hospital, intubated, with a head CT demonstrating a 3.7 × 5.2 × 5 cm left frontal intracerebral hemorrhage (ICH) with extension into the bilateral lateral ventricles, third and fourth ventricles (Figure 3(a)). The patient was Hunt and Hess grade IV + 1, Fisher grade IV. Urine drug screen was positive for meth (records from another hospital). On arrival, her vitals were as follows: blood pressure 143/67 mmHg, heart rate 61 beats/min, respiratory rate 18 breath/min, and Temperature 37.4°C, and GCS was 11T with left-sided hemiparesis. Emergent left craniectomy was done for evacuation of the intraparenchymal hemorrhage and ventriculostomy tube placement. Postoperative formal angiogram, less than 3 weeks from her previous cerebral angiogram, showed that the previously identified fusiform dilations associated with the right pericallosal and callosomarginal bifurcation segment now had a small saccular component with a neck of 5 mm and a height of 7 mm (Figure 2(b)). The previous periophthalmic, basilar tip and left frontopolar aneurysms were identified and remained unchanged. She subsequently underwent angiography assisted coiling of the right pericallosal and callosomarginal bifurcation aneurysm (Figure 3(c)). Over the remainder of her hospital stay, she underwent revision of cranioplasty as well as angiogram for pipeline stent of the periophthalmic artery aneurysm. She was downgraded out of the ICU, ambulating with physical therapy with the assistance of a walker and subsequently discharged home.

## 3. Discussion

Nontraumatic SAH is a neurosurgical emergency with a mortality rate as high as 45%, often secondary to aneurysm rupture [12–14]. Clinically, only two-thirds of all survivors regain functional independence, and nearly half of survivors have permanent cognitive deficits [15]. Among the risk factors for aneurysm rupture including location, morphology, family history, active smoking, and female sex, aneurysm growth is a consistent and significant finding [11, 16, 17]. Brinjijki et al. in a recent meta-analysis reported annual rupture rate in growing aneurysms versus a stable aneurysm as 3.1% and 0.1%, respectively [18]. Further, the greatest risk for aneurysm rupture is during rapid periods of growth and therefore such cases should be dealt with caution and close monitoring [19–21].

Amphetamine and its derivative, methamphetamine, are potent sympathomimetics and among the most common illicitly abused drugs. There is ample scientific data to suggest an association between meth abuse and large artery dissections and aneurysm formation over a period of time leading to rupture and SAH [22]. However, rapid growth of aneurysm, within weeks, and associated meth abuse is quite rare. Previously, Chen et al. was the only one to report the fast growth (within 2 weeks) of an aneurysm on a major intracranial vessel in a habitual amphetamine user [23]. Similarly, in our case where the patient struggling with chronic methamphetamine abuse, there was a very rapid aneurysm growth (within 3 weeks) leading to rupture and ICH. The etiology is hypothesized to be meth or cocaine-induced hypertension and tachycardia (sympathetic rush in the body) that leads to progression, evolution, and growth of intra- and extracranial aneurysms [24, 25].

Additionally vasculitis and other vasculopathic changes have also been postulated as major mechanisms for growth and subsequent rupture in meth abusers [4]. Meth is known to have numerous effects on the central nervous system, specifically the cerebral vasculature. Most prominently, pathological studies of cerebral vasculature have demonstrated necrosis of blood vessel walls with the destruction of the elastic and smooth muscle layer, without leukocytic infiltration of the blood vessel walls, commonly termed necrotizing angiitis or meth arteritis [3]. Chen et al. found much fibrotic adhesion and some fibrinoid necrotic material covering the aneurysm in their reported case [23]. Additionally, blood-brain barrier disruption [26], changes in cerebral perfusion [27], depletion of dopamine and serotonin [28], and cortical grey and white matter loss [29] have all been observed. These changes are seen in both binge and chronic users. Binge methamphetamine use is associated with compromised global and regional blood flow, likely representing severe and enduring neural toxicity of monoaminergic neurotransmitter systems in the brain, eventually producing a pattern of hypoperfusion [29].

Together with the CNS changes observed, transient and extreme increases in sympathetic output with blood pressure elevation can precipitate intracerebral hemorrhage either alone or in association with an underlying vascular pathology such as an aneurysm or vasculitis. A study comprising 30 patients by Ho et al. found all cases of SAH in a patient with methamphetamine abuse were aneurysmal with the majority of aneurysms located in the anterior circulation [30]. Further, numerous studies have demonstrated poor outcomes in patients with meth abuse and ruptured aneurysms when compared to age-matched controls [31, 32] and younger populations of illicit drug users are usually more prone to these devastating events [4, 6, 7].

In our opinion, this case corroborates the acute and chronic cerebrovascular repercussions associated with methamphetamine binge and abuse. In this case rapid growth of the aneurysm leads to rupture and intraparenchymal hemorrhage requiring emergent craniectomy and evacuation. Careful consideration should be given to the rapid development of aneurysms in this patient population as they are at higher risk of rupture. Meth use has been implicated in the growth and rupture of aneurysms, identifying opportunities for early intervention and potential benefit. It is necessary to identify this group of patients early because numerous studies have demonstrated poor outcomes in patients with meth abuse and ruptured aneurysms when compared to age-matched controls, making it imperative to prevent rupture when possible through endovascular or surgical interventions [31, 32]. Based on our experience, we anticipate vigilant monitoring of aneurysms and aggressive strategies treatment in patients with current or previous meth abuse.

## 4. Conclusion

This case highlights the risk of rapid aneurysm growth and rupture as well as the effects of meth on the CNS and cerebral vasculature that may contribute to the higher incidence of SAH in this patient population. The outcome after a ruptured aneurysm in this population is worse than age-matched controls, necessitating close follow-up, and a consideration for more aggressive treatment with endovascular embolization or open surgical clipping after aneurysms are found in patients with methamphetamine abuse. The possibility of a rapid drug-induced pseudoaneurysm should also be considered when faced with intracerebral or subarachnoid hemorrhage in young patients.

## Abbreviations

SAH: Subarachnoid Hemorrhage
Meth: Methamphetamine
ATS: Amphetamine Like Substances
ER: Emergency Room
CNS: Central Nervous system
CT: Computed Tomography
CT-A: Computer Tomographic Angiography
GCS: Glasgow Coma Scale
EVD: Extraventricular Drain
ICU: Intensive Care Unit
ICA: Internal Carotid Artery
CNS: Central nervous system
ICH: Intracerebral hemorrhage.
